# Comparison of IgG4 assays using whole parasite extract and BmR1 recombinant antigen in determining antibody prevalence in brugian filariasis

**DOI:** 10.1186/1475-2883-3-8

**Published:** 2004-08-12

**Authors:** Rahmah Noordin, Sitti Wahyuni, Andarias Mangali, Lim Boon Huat, Maria Yazdanbakhsh, Erliyani Sartono

**Affiliations:** 1 Institute for Research in Molecular Medicine, Universiti Sains Malaysia, 11800 Penang, Malaysia; 2 Dept. of Parasitology, Hasanuddin University, Jalan Perintis Kemerdekaan KM 10 Tamalanrea 90245, Makassar, Indonesia; 3 Department of Parasitology, Leiden University Medical Centre, P.O Box 9600, 2300 RC, Leiden, The Netherlands

## Abstract

**Background:**

*Brugia malayi *is endemic in several Asian countries with the highest prevalence in Indonesia. Determination of prevalence of lymphatic filariasis by serology has been performed by various investigators using different kinds of antigen (either soluble worm antigen preparations or recombinant antigens). This investigation compared the data obtained from IgG4 assays using two different kinds of antigen in a study on prevalence of antibodies to *B. malayi*.

**Methods:**

Serum samples from a transmigrant population and life long residents previously tested with IgG4 assay using soluble worm antigen (SWA-ELISA), were retested with an IgG4 assay that employs BmR1 recombinant antigen (BmR1 dipstick [Brugia Rapid™]). The results obtained with the two antigens were compared, using Pearson chi-square and McNemar test.

**Results:**

There were similarities and differences in the results obtained using the two kinds of antigen (SWA and BmR1). Similarities included the observation that assays using both antigens demonstrated an increasing prevalence of IgG4 antibodies in the transmigrant population with increasing exposure to the infection, and by six years living in the area, antibody prevalence was similar to that of life-long residents. With regards to differences, of significance is the demonstration of similar antibody prevalence in adults and children by BmR1 dipstick whereas by SWA-ELISA the antibody prevalence in adults was higher than in children.

**Conclusions:**

Results and conclusions made from investigations of prevalence of anti-filarial IgG4 antibody in a population would be affected by the assay employed in the study.

## Background

Lymphatic filariasis affects approximately 120 million people worldwide. Ten percent of these infections are attributed to *Brugia malayi *and *Brugia timori *[1]. Thick blood smear examination is the routine parasitological method used for diagnosis and prevalence studies in the *Brugia *endemic countries of Malaysia and Indonesia [2,3]. This diagnostic method depends on the detection of microfilariae in the peripheral blood, and due to the nocturnal periodicity of microfilaremia in these areas, requires nighttime collection and survey, which is often unpopular with the local population. Furthermore, this method is relatively insensitive [4] and difficult to perform accurately and with consistency in field situations.

Conversely, serological diagnostic methods exhibit better sensitivity than detection of microfilaria by thick blood smear, allow the detection of amicrofilaraemic infections among "endemic normals" and afford daytime finger-prick blood sampling (thus overcoming the inconveniences associated with night blood sampling, thereby encouraging greater cooperation with the local population and facilitate field work) [5].

However, reports on antigen detection test for brugian filariasis have not demonstrated high levels of sensitivity [6,7]. Thus in the absence of a good antigen detection test for *Brugia *infection, anti-filarial IgG4 assay may be the next best alternative for detection of brugian filariasis [8]. Anti-filarial IgG4 levels have been demonstrated to be elevated in active filarial infection [9-13] and decline post-treatment [14-17]. Detection of anti-filarial IgG4 antibodies has also been used for epidemiological assessment of filariasis [13,18,19].

Studies assessing antibody prevalence of lymphatic filariasis have employed assays that use either soluble worm antigens or recombinant antigens [12,13,18-21]. These antigens may not bind to the same set of anti-filarial antibodies and probably display different cross-reactivities to antibodies against other infections. Thus differences in the antigens employed may be expected to affect the results of antibody prevalence studies. Therefore, the present study aimed to make direct comparison of two antigens i.e. soluble adult worm antigen (SWA) and a recombinant antigen (BmR1), in IgG4 assays on the same set of serum samples.

Previously, an ELISA employing soluble adult worm antigen (SWA-ELISA) had been performed to determine prevalence of anti-filarial IgG4 antibodies on a set of serum samples from Indonesia. These samples were collected from:

1. A transmigrant population that migrated from a non-filarial endemic region to an area endemic for Brugian filariasis and;

2. Life-long residents of the Brugian endemic area [22].

In the present study a rapid test based on *B. malayi *recombinant antigen (BmR1 dipstick [Brugia Rapid™]) and detection of IgG4 antibodies were evaluated using the same set of serum samples. The BmR1 dipstick test has previously been shown to be highly specific and sensitive for the detection of brugian filariasis. In a study involving four international laboratories, the BmR1 dipstick was found to be 93% sensitive and 100% specific when tested with 535 serum samples from patients with various infections and healthy controls [8]. In another multicenter validation study, 97% sensitivity and 99% specificity were recorded when the BmR1 dipstick was tested with 753 serum samples [23].

The present study demonstrated that interpretations of some aspects of the seroepidemiology of filarial infection are affected by the kind of antigen employed in the assay. Thus this study, which utilized the same set of serum samples on assays using two kinds of antigens, highlights the role of the kind of antigen employed in the comparison of results of prevalence studies.

## Materials and methods

### Sera and study population

The details on the sera and study population are as described previously [22]. Briefly, serum samples were collected cross-sectionally from a total of 247 transmigrants and 133 life-long residents (LLR) from Budong-budong, a district of Mamuju Regency in South-Sulawesi, Indonesia, which is endemic for nocturnal-periodic *B. malayi *[24,25]. The samples from the transmigrant population are valuable as they could help determine the pattern of acquisition of infection with increasing length of exposure to Brugian filariasis. The transmigrant population had traveled to their new homesteads in groups; they came from the same village or region in Bali or Lesser Sunda islands as part of the government-sponsored relocation programme. Each year a new settlement was founded close to the former one (between 10 and 20 kilometers) which accommodated groups of transmigrants from 2–3 different regions together with migrants from Polmas, an over-populated area in South Sulawesi, to promote integration of different tribes. Transmigrants were grouped together according to the year of arrival in the new settlement. A total of 6 transmigrant units, settled between several months and 6 years prior to the survey, were included in the study together with 2 villages of indigenous Sulawesians (LLR), which were situated closely to the transmigrant areas. Those aged ≤ 15 years were classified as children, while adults were classified as those aged 16 years and older. The mean age of children in transmigrant population and LLR population was 10.2 years and that of adults was 32.6 years.

### Soluble worm antigen (SWA)

Adult *B. malayi *worms were purchased from TRS labs, Athens, Georgia, USA. Female worms were freeze dried, ground to powder, dissolved in phosphate buffered saline (PBS), homogenized and slowly stirred overnight at 4°C. The protein concentration was determined by 2,2'-biquinoline-4,4'-dicarboxylic acid disodium salt hydrate (BCA) method before storage at -20°C.

### BmR1 dipstick

This BmR1 dipstick (Brugia Rapid™) was performed as described previously and according to the instructions of the manufacturer [16, Malaysian Bio Diagnostics Research Sdn. Bhd., Bangi, Selangor, Malaysia]. The BmR1 recombinant antigen was expressed from *B*m*17DIII *DNA sequence, GenBank accession no. **AF225296.** Southern blot hybridization assays performed on cDNA libraries of L3, L4, mf, adult male and adult female *B. malayi *demonstrated that the DNA sequence is present in all of the five kinds of libraries (Rahmah *et al*., unpublished data). Preliminary immunohistological studies suggest that the expressed antigen is found in the epithelial membranes of the adult female uterus (Rahmah *et al*., unpublished data).

### Statistical Analysis

The results obtained with BmR1 dipstick were compared to the results obtained previously with SWA-ELISA. The similarities and differences in the results were analyzed by comparing proportion of related samples using McNemar test; and comparing proportions of unrelated samples by using Pearson Chi-square (if indicated Fischer exact test was used instead).

## Results and Discussion

**Figure 1:**
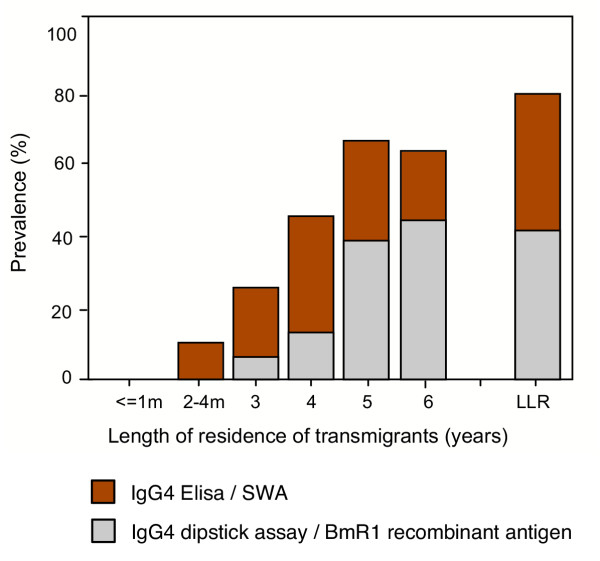
IgG4 antibody prevalence in transmigrant population who resided in *B. malayi *endemic areas for various lengths of time and in life-long residents (LLR), as determined by BmR1 dipstick and SWA-ELISA.

Figure 1 shows the antibody prevalence to *B. malayi*, as determined by BmR1 dipstick assay and SWA-ELISA, in the transmigrant population who had resided for various lengths of time in the endemic area and the antibody prevalence in the LLR population. The first detection of IgG4 antibody by the BmR1 dipstick was recorded at 3 years post-exposure, the antibody prevalence increased from 0% in the new arrivals (≤ 1 month and 2–4 months) to 7.4%, 11.1%, 39.1% and 42 % in populations exposed to the infection for 3, 4, 5 and 6 years respectively. Comparison of IgG4 assays using the two kinds of antigen demonstrated similarities in three areas. First, using both SWA-ELISA and BmR1 dipstick, the total prevalence of specific IgG4 in the transmigrant population was found to increase with increasing length of residence in the endemic area. Thus the BmR1 dipstick test confirmed the previously reported finding which demonstrated that the development of anti-filarial IgG4 correlated with the duration of exposure in previously unexposed population [22]. Second, after a period of 5–6 years of being exposed to the filaria infection, anti-filarial IgG4 prevalence by BmR1 dipstick in the transmigrant population (42%) was comparable (p = 0.763) to the antibody prevalence in the LLR population (39.8%); this finding was also previously reported with SWA-ELISA [22]. Thus the results with BmR1 dipstick is in agreement with the previous finding that approximately 5–6 years of exposure is required for the anti-filarial IgG4 in the transmigrant population to reach levels comparable to life-long residents. This is also reported to be the period needed for the detection of microfilaria in the peripheral blood [22]. Third, the overall prevalence of anti-filarial IgG4 was found to be higher in males than in females, by both BmR1 dipstick (p = 0.019) and by SWA-ELISA (p = 0.001).

Differences between the two kinds of antigen were also observed (Figure 1). First, except for year 5 transmigrants (p = 0.146), the percent antibody prevalence recorded in the transmigrant population and in the LLR population by BmR1 dipstick were significantly lower than that detected by the SWA-ELISA i.e. year 3, p = 0.00; year 4, p = 0.00; year 6, p = 0.041; LLR, p = 0.00. Second, using SWA-ELISA, anti-filarial IgG4 was first detected at 2–4 months post-exposure in the population of transmigrants, while using BmR1 dipstick the first detection of IgG4 antibody was recorded after three years of residence in the endemic area.

**Figure 2:**
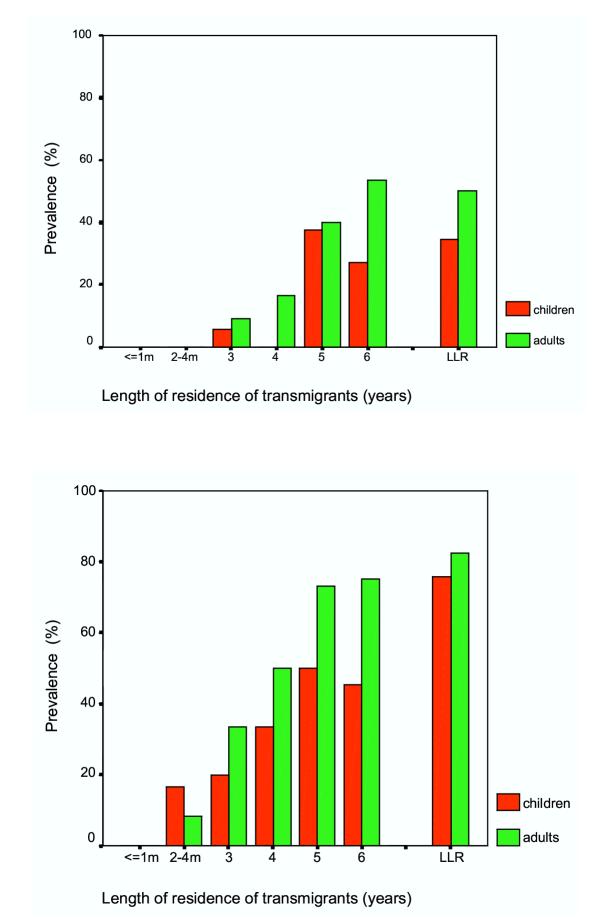
IgG4 antibody prevalence in transmigrant adult and children populations who resided in *B. malayi *endemic areas for various lengths of time and in life-long residents (LLR). **a **(top) IgG4 antibody prevalence as determined by BmR1 dipstick assay. **b **(bottom) IgG4 antibody prevalence as determined by SWA-ELISA. (Note: Previously published in Parasitology 2001; 122, pg. 636 [Reproduced with permission]).

Figure 2a and 2b shows the IgG4 antibody prevalence among children and adult populations as determined by BmR1 dipstick and SWA-ELISA respectively. There was no significant difference detected in antibody prevalence between adults and children in the transmigrant population and in the LLR population when BmR1 dipstick was used (year 3, p = 0.594; year 4, p = 0.066; year 5, p = 0.907; year 6, p = 0.061; LLR, p = 0.074). Using SWA-ELISA, except for early transmigrant settlers (2–4 months residents), the IgG4 prevalence in the transmigrant population was reported to be significantly higher in adults than in children [22]; however in the LLR population, antibody prevalence in children was not significantly higher than in adults (p = 0.316).

Thus the BmR1 dipstick test demonstrated that the establishment of anti-filarial IgG4 in the children and adults of the transmigrant population occur at comparable rates; this is not in agreement with the previous finding, using SWA-ELISA, that demonstrated IgG4 was established more rapidly in the adult population than in children [22]. The above observations may be due to a mixture of filarial antigens in SWA, some of which may recognize antibodies produced by exposed but not infected individuals, and/or by individuals who have cleared the infection either due to treatment or spontaneous death of worms. Although IgG4 detection significantly increases the specificity of antibody assays in brugian filariasis [12] the specificity of parasite extract-based assays and recombinant antigen-based assays may not be the same. In a recent study in a non-filaria endemic area in Brazil, the presence of *Strongyloides *antibody responses was found to be associated with higher antifilarial IgG4 responses in assay that uses crude filaria extract as compared to assay that uses *B. malayi *Bm14 recombinant antigen [26].

The discrepancy between the results of SWA-ELISA and BmR1 dipstick may also be partly due to the greater sensitivity of the former as compared to the latter. Out of 120 (of 381) discrepant results, 111 were positive by SWA-ELISA but negative by BmR1 dipstick. Out of these 111 samples, 38 had low ELISA titers (cut-off value > 4.02 but < 4.5). If the stringency of the cut-off value of the SWA-ELISA is increased from 4.02 to 4.5, then the discrepancy can be considerably reduced to 88 (of 381), with 73 positive by SWA-ELISA but negative by BmR1 dipstick. On the other hand, since BmR1 dipstick test has been reported to be highly specific [8,23], it is unclear why there were eight individuals who were positive by BmR1 dipstick but negative by SWA-ELISA.

Due to the problem of maintaining the antigenicity of the BmR1 antigen when shipped from Malaysia to Netherlands, the dipstick (immunochromatography) assay, which can be transported at room temperature, was employed to test the BmR1 antigen. However the difference in the assay formats is unlikely to be the reason for the lower overall antibody prevalence levels seen with the latter. This is because we have previously shown that ELISA using BmR1 was less sensitive (*albeit *equally specific) than BmR1 dipstick test [27] in detecting *B. malayi *infection. Thus in this study if the ELISA format had been used to determine prevalence of IgG4 antibodies to BmR1 instead of the dipstick format, the SWA-ELISA would still be detecting significantly more positives than the BmR1-ELISA.

Differences between the two antigens were also observed in the pattern of IgG4 antibody prevalence in children (Figure 2a &2b). Using BmR1 dipstick, positivity of the test in children was first demonstrated at 3 years (2 of 35, 5.7%), followed by no positive child detected at year 4 (0 of 17), positive antibody prevalence at year 5 (3 of 8; 37.5%) which is significantly greater than at year 3 (p = 0.011). This is followed by a nonsignificant decrease (p = 0.589) in antibody prevalence by year 6 (6 of 22; 27.3%). IgG4 prevalence at year 6 and LLR (30 of 87; 34.5%) was also found to be not significantly different (p = 0.542). After year 4, the IgG4 prevalence in children seemed to achieve a stable level that was similar to the antibody prevalence in the LLR population children. In adults, infection was also initially detected at year 3 and there appeared to be a pattern of increasing antibody prevalence with increasing time of residence (9.1% at 3 years; 16.7% at year 4, 40% at year 5; 53.6 % at year 6). Furthermore, the detection rate at year 6 was not significantly different (p = 0.766) from that seen in the LLR population adults (50%). Thus using the BmR1 dipstick, the increasing total IgG4 prevalence with exposure to brugian filariasis was mostly due to the increasing positive cases in the adult population. It is tempting to speculate that the differences in the pattern observed in children and adults are due to the greater rate of aborted infections and/or spontaneous clearance of the infection in children than in adults. The overall lower worm burden in children, due to physiological differences, may enable higher rate of spontaneous clearance of infections in children than in adults. The difference in physiology between adults and children has been demonstrated by the greater number of natural killer cells, and T-and B-lymphocytes in children as compared to middle aged people [28]. In addition CD4/CD8 T-cells have also been reported to decline from a young age onwards [29,30]. However, since this is a cross-sectional study in which data for each period of residence were obtained from different groups of individuals, this hypothesis could not be confirmed.

Conversely, results of the SWA-ELISA demonstrated initial antibody positivity in children at 2–4 months, followed by increasing antibody prevalence with time of residence. IgG4 prevalence at year 6 was found to be significantly lower than the antibody prevalence in children of the LLR population (p = 0.007). In the adult population, except for the earlier initial detection, a similar pattern was observed with the results of the BmR1 dipstick i.e. increasing rate of antibody prevalence in the transmigrants with exposure; and the IgG4 prevalence at year 6 is not significantly different (p = 0.430) than the antibody prevalence in the LLR adult population.

## Conclusions

This study highlights that assays using both BmR1 and SWA antigens demonstrate an increasing prevalence of specific IgG4 antibodies in the transmigrant population with increasing length of residence in an area endemic for brugian filariasis, and, by six years residency that the antibody prevalence was similar to that observed in the LLR population. This study also documented three main differences in results derived from assays using two antigens i.e.

1. Earlier detection and higher rate of antibody prevalence by SWA-ELISA as compared to the results demonstrated by BmR1 dipstick;

2. Similar rate of acquisition of antibody prevalence in children and adults by BmR1 dipstick; whereas by SWA-ELISA adults were found to become antibody positive faster than in children;

3. By BmR1 dipstick the increasing total prevalence of IgG4 with exposure was primarily due to the adult population, whereas by SWA-ELISA this was attributed to both children and adult populations.

This study demonstrates that some aspects of seroepidemiology of *Brugia malayi *infection may vary with the kind of antigen used in the assay. Thus comparison of results of different studies must take into account the kind of antigen employed, especially if one study uses native antigen and another uses recombinant antigen. It would be interesting to compare seroepidemiological data of IgG4 assays using two different recombinant antigens on the same population.

## Competing interests

Rahmah Noordin is the inventor of the commercialized BmR1 dipstick test (Brugia Rapid™)

## Authors' contributions

RN drafted the paper and supplied the BmR1 dipstick test; SW & AM supplied the sera and edited the paper; LBH performed most of the statistical analysis; ES and MY conceived the study and contributed significantly in editing of the paper: In addition ES performed the BmR1 dipstick test and some statistical analysis. All authors read and approved the final manuscript.
